# Self-assembled and intercalated film of reduced graphene oxide for a novel vacuum pressure sensor

**DOI:** 10.1038/srep38830

**Published:** 2016-12-15

**Authors:** Sung Il Ahn, Jura Jung, Yongwoo Kim, Yujin Lee, Kukjoo Kim, Seong Eui Lee, Sungyun Kim, Kyeong-Keun Choi

**Affiliations:** 1 Department of Engineering in Energy and Applied Chemistry Silla University, Busan 617-736, Republic of Korea; 2 Department of Electrical Engineering Korea Advanced Institute of Science and Technology (KAIST), 291 Daehak-ro, Yuseong-gu, Daejeon 305-701, Republic of Korea; 3 Advanced Materials Engineering Korea Polytechnic University, Jungwang dong Shihung 429-793, Republic of Korea; 4 Institute of NT.IT fusion technology, Ajou university, Worldcup ro 260, Youngtong gu Suwon 16499, Republic of Korea; 5 National Institute for Nanomaterials Technology (NINT) San 31, Hyoja-Dong, Nam-Gu, Pohang 790-784, Republic of Korea

## Abstract

We report a new method for measuring vacuum pressures using Van der Waals (VDW) interactions between reduced graphene oxide (RGO) sheets. For this purpose, we utilized a reaction-based self-assembly process to fabricate various intercalated RGO (i-RGO) films, and monitored their electrical behavior with changing pressure and temperature. Pumping to remove gas from a vacuum chamber produced a decrease in the sheet resistance of i-RGO. With further pumping, distinctly different sheet resistance behaivors were observed depending on the measurement temperature. With increasing vacuum pressure, the resistance increased at 100 °C, whereas it decreased at 30 °C. Two types of VDW interactions are proposed to explain these features: a local VDW interaction between RGO sheets that resulted in V-shaped curves of sheet resistance with pressure changes and broad VDW interactions that occur between RGO sheets when the elastic force required to bend carbon clusters on an RGO sheet exceeds their vibrational energy at low temperatures. On the basis of the results, we propose that the resistance behavior of i-RGO as a function of vacuum pressure can be interpreted as the sum of the two different VDW interactions.

Much research on graphene and related materials has been highly focused on sensor applications. Such applications include molecular sensors^1^, radiation sensors^2^, strain sensors^3^, temperature sensors^4^, various biosensors^5^, and pressure sensors^6^,^7^,^8^,^9^,^10^,^11^,^12^,^13^. Graphene-based pressure sensors mostly consists of Piezoresistivity-type sensors that determine the strain produced in graphene by an external pressure and can thus precisely measure pressures much greater than atmospheric levels^6^,^7^,^8^,^9^. Only a few studies on sensors for measuring vacuum pressures have been reported^10^,^11^,^12^,^13^. For example, graphene on a silicon nitride membrane perforated by a periodic array of micro-through-holes exhibited pressure readings with high linearity^10^. A direct electrical readout of pressure via strain transduction was demonstrated using piezoresistive effects in a suspended graphene membrane^11^. In addition, a graphene squeeze-film sensor displayed pressure dependence of the resonant frequency produced by compression of the surrounding gas^12^. However, such sensors have rarely been used to measure vacuum pressures below 1 torr. Consequently, no basic principles for using graphene to measure vacuum pressures below 1 torr have been established, despite their wide applicability in vacuum gauges. Accurate vacuum measurements are crucial for determining product quality in modern industrial settings and in vacuum-dependent scientific research.

Recently, we established a new method for measuring vacuum pressures using van der Waals (VDW) interactions between intercalated RGO (i-RGO) sheets (See Figure S1 and S2). The principle for using i-RGO as a vacuum sensor is described in Fig. 1a. The optimal distance for exhibiting VDW attraction is calculated as 2.5 × σ (a Lennard–Jones parameters equal to 0.34 nm for graphite), which corresponds to d = 0.85 nm^14^. This value indicates that attractive forces should exist between most RGO sheets in a film. However, the gas molecules filling void spaces in the film prevent an RGO sheet from approaching adjacent sheets. As the gases are pumped out, RGO sheets become close to the ideal distance for maximum VDW interaction. Under these circumstances, we speculate that two types of VDW interactions occur on i-RGO. The first type involves a local VDW (LVW) interaction (See Fig. 1a) between RGO sheets in a film, which produces V-shaped curves of sheet resistance with changes in pressure (also refer to Figure S3 for a theoretical description of LVW). The second type consists of wide VDW (WVW) interactions (See Fig. 1b) between RGO sheets that occur when the elastic force required to bend carbon clusters in an RGO sheet exceeds their vibrational energy at low temperatures. The electrical conductivity is directly related to the number of charge carriers and their drift mobilities. If RGO sheets in the i-RGO samples have similar degrees of reduction, the pattern of the i-RGO sheet resistance versus pressure can be explained simply by charge carrier mobility. The conduction paths illustrated in Fig. 1a and b indicate that the charge carrier mobility decreases in the case of LVW interactions owing to the formation of potential barriers by elastic deformation and increases in the case of WVW interactions owing to the reduction of potential barriers by the decreased distance between RGO sheets. Elastic deformation of carbons caused by attractive VDW interactions has been reported in several studies on carbon nanotube (CNT)^14^,^15^,^16^,^17^. For example, VDW interactions between CNTs or between a CNT and a substrate cause radial deformation of CNTs^15^,^16^, as shown in Fig. 1c, although the elastic deformation extent is limited by the number of carbon atoms composing the ring.

We attempted to verify these two types of interactions by controlling the amount of intercalated molecules between RGO sheets since the size of carbon clusters to be deflected is directly related to their elastic force and decreases as the amount of intercalation increases. A recently reported reaction-based self-assembly (RSA, see Figure S4) method was determined to be suitable for preparing i-RGO films for the above purpose^18^. In addition, resistance of the films is expected to be controllable for sensor applications by controlling the amount of intercalation. Since this research ultimately aims to miniaturize a vacuum sensor for the development of wireless sensors requiring ultra-low power consumption, the resistance of the i-RGO film is considered to be one of the most important factors for such applications. In this study, RGO films containing various amounts of a water-soluble polymer, poly-vinyl-alcohol (PVA), were prepared using the RSA method. They were then characterized and their electrical behavior was examined under changing vacuum pressure to determine their potential sensor applications.

## Results and Discussion

**Figure 2:**
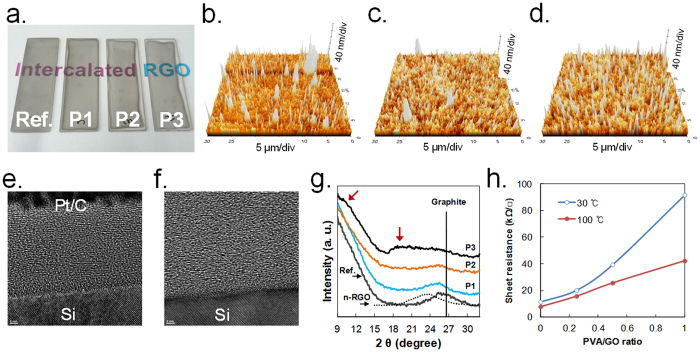
Characterization of an i-RGO film with various PVA contents (0 (Ref.), 0.25 (P1), 0.5 (P2), and 1 (P3) to GO content in weight ratio); (**a**) A photograph of i-RGO samples on a 7 × 5 cm piece of glass, AFM images of (**b**) P1, (c) P2, and (**d**) P3. TEM cross-sectional images of (**e**) Ref. and (**f**) P3, (**g**) XRD spectra of i-RGO samples after heat-treatment at 400 °C for 1 h, (**h**) Sheet resistances of the samples at 30 °C and 100 °C. Note that the n-RGO sample in (**g**) was formed by spin-coating method and heat-treating at 400 °C for 1 h.

The RSA processes were conducted with various GO mixtures containing hydrazine and various PVA content. Above a PVA/GO weight ratio of 1, i-RGO films were not well formed by the RSA method, whereas we obtained clean films of i-RGO below a PVA/GO weight ratio of 1. Partial contraction of the i-RGO films are observed, which increased as the PVA content was increased (See Fig. 2a). All the i-RGO samples had approximately around 45% transmittance at 550 nm. The FT-IR spectra of i-RGO indicate that most of the oxygen groups disappeared during the RSA process (See Figure S4). Many conical-shaped bumps can be seen in the AFM image of i-RGO (See from Fig. 2b to d), which are uniformly distributed on the i-RGO surface and become enlarged from 40 to 80 μm in height and 0.2 to 0.6 μm in diameter as the PVA content is increased. On the basis of this result, we infer that intercalation of PVA occurs uniformly during the RSA process, although the reasons for the formation of the bumps are not clear. The RGO sheets in the Ref. RGO film display slightly wavy, regular patterns in a cross-section image, whereas such regular patterns are not clearly observed in i-RGO (P3), likely owing to the presence of PVA decomposition residues of PVA (Refer to Fig. 2e and f). The XRD spectra in Fig. 2g contains broad peaks that develop at approximately 10° and 19° as the PVA content increases. Other characterization results for the i-RGO samples are shown in Figure S4. The sheet resistance of the sample also increases as the PVA content is increased (See Fig. 2h). Seemingly, the increase of resistance of RGO appears to be easily achieved by controlling the degree of GO reduction; however, it was found that most of the GO samples, had similar conductivities when they were reduced by hydrazine and then thermally treated above 180 °C, irrespective of hydrazine content^19^. The RSA process yielded similar results because intermediates of the GO with hydrazine and/or some of the unreacted hydrazine were involved in the RSA process. In contrast, the result in Fig. 2g demonstrates that the resistance can be easily controlled by varying the PVA content.

**Figure 3:**
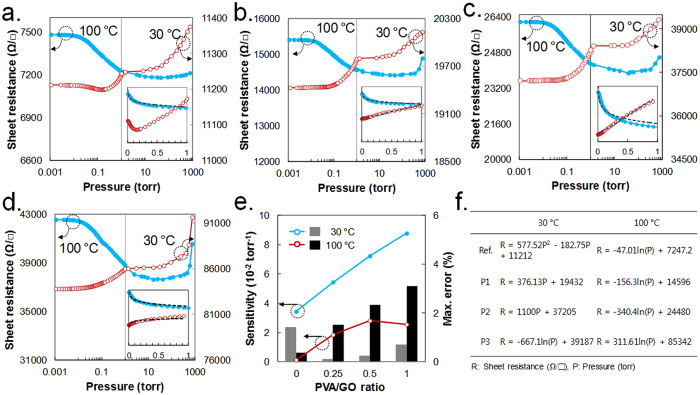
Sheet resistances of i-RGO samples with increasing pressure from 10^−3^ torr to ambient air pressure at 30 °C and 100 °C for (**a**) Ref., (**b**) P1, (**c**) P2, and (**d**) P3. (**e**) Sample sensitivities calculated as ΔR/R_max_·P. Here, P = |P_max_ − P_min_| and ΔR = |R_max_ − R_min_|, where R_max_ and R_min_ are the maximum and minimum values of sheet resistance in the pressure range between 0.001 and 1 torr, respectively (except Ref. at 30 °C, where R_min_ is the minimum sheet resistance in Fig. 3a) and maximum errors in pressure readings calculated using the ideal curves in the inset of the enlarged sheet resistance graph on a linear scale in the relevant pressure range, (**f**) A table of curve-fitting equations used for calculations of sensitivities and errors in (**e**).

We measured the sheet resistance of the i-RGO films as a function of vacuum pressure using a four-point probe placed in a vacuum chamber at 30 °C and 100 °C after baking at 100 °C for 30 min. At low vacuum pressures between 1 and 760 torr at 30 °C, or around 10 and 760 torr at 100 °C, all the i-RGO samples exhibited a linear decrease of sheet resistance (See Fig. 3a–d). The rate of change in resistance in this pressure range is very low compared with those in the spin-coated RGO films studied in the preceding works (See Figure S1). The peak located near the graphite peak in the XRD spectrum indicates that the Ref. RGO prepared by the RSA method has a smaller d-spacing than that of RGO formed by spin coating. In addition, the sheet resistance of the Ref. RGO is several times lower than that of the spin-coated RGO. These results suggest that the RGO films formed by RSA contain less void space and exhibit a small change in resistance in the targeted pressure range. Below approximately 1 torr, the plotted sheet resistances results appears distinctly different for different measuring temperatures. At 100 °C, the resistance increases as the vacuum pressure is increased, whereas at 30 °C, the resistance decreases except for in the reference sample. As described in the introduction, the VDW attraction force appears below a distance of 2.5 × σ (a Lennard–Jones parameter equal to 0.34 nm for graphite) and increases exponentially as the distance decreases. Therefore, the vibrational energy of RGO positively influences the VDW attractive interaction between carbon clusters or entire carbons on adjacent RGO sheets rather than taking the carbon clusters off. At low temperature, the vertical vibration distance of carbon clusters on the RGO sheets appears to be too small for the clusters to deform by VDW interactions. In contrast, at high temperatures, the VDW attractive interaction appears to occur locally rather than broadly, owing to the large vibration distance of the carbon clusters. The theoretical description based on the vertical motions of carbon on an RGO sheet is consistent with the experimental results at high temperature (See Figure S3). This result indicates two important features for the application of RGO to vacuum pressure sensors. An RGO sensor with a non-linear (parabolic curve) response requires a complex combination of circuits or sensors to measure vacuum pressures. However, a unidirectional response (increase or decrease in resistance) with changes in vacuum pressures allows a simplified module to be used for the RGO sensor. More importantly, the sample exhibits unidirectional response in the sheet resistance at low temperature (30 °C).

For the pressure range between 10^−3^ and 1 torr, the sensitivity of each sample was calculated using ΔR/R_max_·P. Here, P = |P_max_ − P_min_| and ΔR = |R_max_ − R_min_|, where R_max_ and R_min_ are the maximum and minimum sheet resistance in the pressure range, respectively, except for Ref. at 30 °C, for which R_min_ is the minimum sheet resistance in Fig. 3a. As shown in Fig. 3e, the sensitivity of i-RGO is over ten times higher at 30 °C and twice as high sensitivity at 100 °C in comparison with that of the Ref. RGO. The maximum errors in the pressure readings are 0.1% at 30 °C and 1.5% at 100 °C for P1, 0.26% at 30 °C and 2.3% at 100 °C for P2, and 0.7% at 30 °C and 3.1% at 100 °C for P3. The equation used to calculate sensitivities and errors for each sample is given in Fig. 3f. The sensitivity of i-RGO (P3) can be compared with results from previous studies on graphene-based vacuum pressure sensors. As shown in Table 1, the i-RGO sensor is estimated to have a sensitivity similar to the that of most sensitive piezoresistive sensor reported previously. For comparison, the dimensions of the i-RGO sensor in Table 1 were determined by the active area of the test device in Figure S5 after dividing by a correction factor calculated from the sensitivity ratio of the i-RGO film to the i-RGO device.

A similar experiment for a higher vacuum pressure range between 1 × 10^–3^ and approximately 1 × 10^–6^ torr was conducted at low temperature (20 °C) using a high-vacuum chamber (See Figure S6). We found that the RGO sensor was able to detect the maximum vacuum pressure of the chamber, although the pressure reading was not precisely recorded owing to the influence of the hot cathode ion-gauge on the sheet resistance of sample P3.

**Figure 4:**
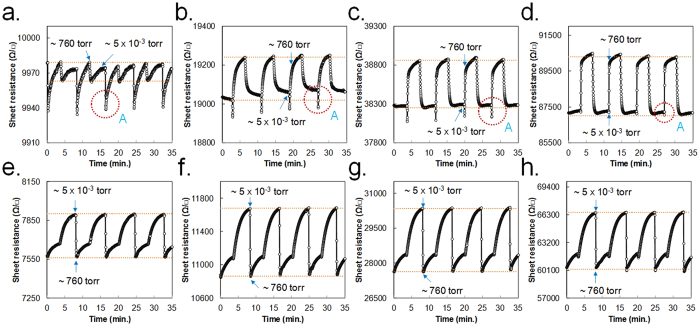
Reproducibility of pressure reading from i-RGO sample in pressure reading at 30 °C for (**a**) Ref., (**b**) P1, (**c**) P2, (**d**) P3, and at 100 °C for (**e**) Ref., (**f**) P1, (**g**) P2, (**h**) P3. Note that the resistance fluctuation in area A is caused by the pressure difference between the test chamber and the RGO sample when the chamber is instantaneously vented.

The reproducibility of the pressure readings for each sample was tested at 30 °C and 100 °C. We depressurized the test chamber to approximately 5 × 10^−3^ torr and then vented it completely. The sheet resistance of the sample was simultaneously recorded at each step. Graphs of the repeat measurements display a regular change in the sheet resistance with time for each sample (See Fig. 4), indicating that the resistance of all i-RGO samples responds steadily and rapidly to pressure changes. Minor fluctuations in the maximum or minimum resistance occurred at 30 °C, likely because of the slow rate of gas diffusion. In this test, we can observe that the sample exhibits an interesting response to pressure at 30 °C. The points marked A in Fig. 4a–d indicate a sudden drop in the sheet resistance of each sample in response to a rapid change in pressure. These features are apparently caused by the pressure difference inside and outside of the film that occurs when the pressure leak valve is opened instantaneously. Because the external pressure is larger than the pressure inside the film at the moment of the sudden pressure leak, the RGO film appears to become compressed owing to the pressure difference. This decreases the distance between RGO sheets and leads to an increase in sheet-to-sheet flow of charge carriers in the film. This result suggests that the decrease in sheet-resistance of the i-RGO films in Fig. 3, below 1 torr at 30 °C, is also related to the decrease in distance between RGO sheets. Considering that the decrease in distance is dominantly caused by VDW interactions if no external influences are present, we can deduce that WVW interactions exist between RGO sheets below 1 torr at 30 °C. According to the report mentioned in the introduction, elastic deformation of a CNT is limited by the number of carbon atoms composing the ring. Theoretical calculations reveal that CNTs containing fewer than 10 carbon atoms in the ring maintained their circular configuration as a result of VDW interactions^16^. If the elastic forces of the carbon clusters on the RGO sheet are sufficiently strong to restrict thermal vibrations, they can retain their configuration in a manner similar to CNTs. However, unlike in the case of CNTs, it appears that the WVW interaction occurs and reduces the distance between RGO sheets in the present study. This is because the distance between RGO sheets (ranging approximately from 0.42 to 0.35 nm based on the XRD spectrum of P3 in Fig. 2g) is near the range of maximum VDW attractive interaction^8^. Since the intercalate distribution is not completely uniform, we can deduce that the resistance behavior of i-RGO as a function of pressure is determined by the sum of LVW and WVW interactions.

In summary, we introduced an RSA method to intercalate PVA polymer between RGO sheets. Upto a PVA/GO weight ratio of 1, uniform i-RGO films were formed by this method. In addition, the sample resistance was effectively controlled by varying PVA content. The XRD spectra indicated development of void space in the i-RGO samples as the PVA content was increased. This result suggests that intercalation of PVA by the RSA method increases with higher PVA content. From the sheet-resistance behavior of the samples versus pressures below 1 torr, we found that the LVW interaction dominantly occurred at 100 °C owing to the large vibrational energy that allows carbon clusters to become curved on the RGO sheet by VDW interaction. In contrast, at 30 °C, the WVW interaction preferentially occurred over the LVW interaction because the elastic force of the carbon clusters overwhelmed the vibrational energy of the RGO sheets. This resulted in a decrease in the sheet resistance of i-RGO as the degree of vacuum was increased. Compared with other pressure sensors, i-RGO-based sensors potentially offer ultra-thinness, flexibility, high durability, and easy integration into circuits. With better control of the spacing between RGO sheets as well as gas diffusion rates, an i-RGO based sensor that precisely measures pressures ranging from ambient air to a high vacuum can be designed.

## Methods

GO was prepared from synthetic graphite (Aldrich, <20 μm) by a modified Hummers method. The GO mixture was diluted to 0.02 wt%; then, 10 ml of the GO mixture was mixed with 1 ml of various contents of a PVA solution with PVA to GO ratios of 0, 0.25, 0.5, and 1. The samples of the PVA/GO mixture were termed Ref. (for a ratio of 0, normal RGO), P1 (for 0.25), P2 (for 0.5), and P3 (for 1). Then, 6 ml of a 2 wt% aqueous diluted solution of hydrazine (80 wt%, Daejung) was added to the PVA/GO mixture. On a hot-plate with ± 1 °C accuracy at 200 °C, 6 layers of clean wipers were placed to slowly vent water vapor during the RSA process for RGO, and the horizontality of the fabric surface was adjusted using a level with 0.02 mm accuracy. The process temperature was around 50 °C measured at the surface of the venting fabric when the temperature of the hot plate was set to around 75 °C. After the temperature of the fabric reached 50 °C, we placed a glass substrate on it, waited until the temperature of the substrate reached around 30 °C, and then added the final mixture onto the glass substrate in drops until it covered the entire surface. The amount of the reaction mixture was 0.055 ± 0.002 g/cm^2^. Finally, the process environment was isolated by a conically shaped glass cover to protect the reaction area from unexpected air flow, and from water drops that formed by condensation along the inside wall. The self-assembly process was terminated after about 80 min or after the disappearance of a circular mark of water was observed. All samples were thermally treated at 200 °C for 1 h under air and then 400 °C for 1 h under a vacuum. The samples were characterized by Fourier transform infrared spectroscopy (FT-IR; Shimadzu, IRTracer-100), X-ray diffraction (XRD 6000 model, Shimadzu) using Cu Kα radiation at 30 kV and 30 mA, X-ray photoelectron spectroscopy (XPS; VG Scientific), and atomic force microscopy (AFM; Nanowizard, SFM). To examine the sensor activity, we placed the sample on a heating plate in a vacuum chamber with a four-point probe. The probe measured the sheet resistances at 1.67 s intervals as a function of elevated vacuum pressure under a constant pressure leakage of approximately 3 × 10^−3^ torr/min up to around 1 torr. After reaching 1 torr, we manually increased the chamber pressure and measured the sheet resistance of the sample. The vacuum pressure was measured between 1 and 10^−3^ torr by a calibrated capacitor gauge with a maximum error of 0.3%. Prior to measurement, the samples were heat-treated for 30 min at 100 °C. To assess the potential applicability of RGO films to pressure sensors, we performed a reproducibility test. In this test, we depressurized the chamber to around 5 × 10^−3^ torr, vented it completely after 4 min, and maintained the vacuum for 4 min. The sheet resistance of i-RGO films at 30 °C and 100 °C were recorded during several trials of this process.

## Additional Information

**How to cite this article**: Ahn, S. I. *et al*. Self-assembled and intercalated film of reduced graphene oxide for a novel vacuum pressure sensor. *Sci. Rep.* **6**, 38830; doi: 10.1038/srep38830 (2016).

**Publisher's note:** Springer Nature remains neutral with regard to jurisdictional claims in published maps and institutional affiliations.

## Supplementary Material

Supplementary Information

## Figures and Tables

**Figure 1:**
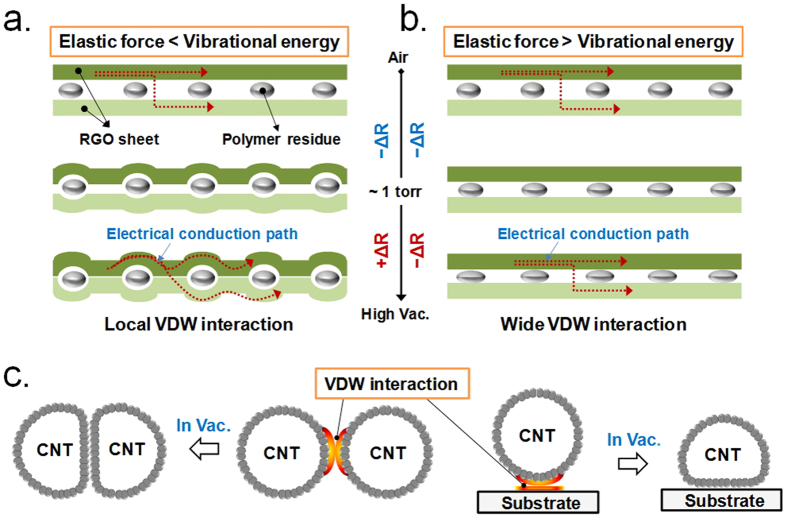
Diagrams illustrating the sensing principle of i-RGO for measuring vacuum pressure; (**a**) LVW interaction between i-RGO sheets in a film at high temperature, leading to an increase in the resistance of the i-RGO film as the vacuum pressure increases in the range below 1 torr, (**b**) WVW interaction between i-RGO sheets in a film at low temperature, leading to a decrease in the resistance of the i-RGO film as the vacuum pressure increases, (**c**) Elastic deformation of CNTs^14^,^15^,^16^,^17^. Note that the elastic deformation of the CNTs is restricted by the number of carbons in their ring.

**Table 1:** Comparison of i-RGO sensitivity with the results of previous studies on graphene-based vacuum pressure sensors.

Type of sensor	Dimensions (μm^2^)	Pressure range (torr)	Sensitivity (torr^−1^)	Ref.
Intercalated RGO film (Van der Waals force)	600 × 600	0.001~11~760	5.7 × 10^−2^3.7 × 10^−5^	P3 at 100 °C (This work)
	600 × 600	0.001~760	7.4 × 10^−5^	P3 at 30 °C (This work)
Graphene on SiN_x_ cavity (Piezoresistivity)	490 × 490	0 (unspecified)~300	3.7 × 10^−5^	Wang *et al*., Nanoscale^10^
Graphene on SiO_2_/Si cavity (Piezoresistivity)	6 × 640	150~760	3.9 × 10^−6^	Smith *et al*., Nano Lett.^11^
Graphene squeeze-film (Resonant frequency)	5 × 15	6~760	3.2 × 10^−4^	Dolleman *et al*., Nano Lett.^12^
Graphene on SiN_x_ cavity (Piezoresistivity)	280 × 280	0 (unspecified)~530	8.9 × 10^−6^	Zhu *et al*., Appl. Phys. Lett.^13^

In this table, i-RGO sample dimensions were determined by the active area of the test device shown in Figure S6 after dividing by a correction factor calculated from the sensitivity ratio of the i-RGO film to the i-RGO device.
