# Supplementary material for: Self-assembled and intercalated film of reduced graphene oxide for a novel vacuum pressure sensor
Source: Sci Rep. 2016 Dec 15;6:38830. doi: 10.1038/srep38830 (PMC5156946; doi:10.1038/srep38830)
Supplement: Supplementary Information [file srep38830-s1.pdf]

Supplementary Information for

Self-assembled and intercalated film of reduced graphene oxide for a novel vacuum pressure sensor

Sung Il Ahn[1*], Jura Jung[1], Yongwoo Kim[1], Yujin Lee[1], Kukjoo Kim[2], Seong Eui Lee[3*], Sungyun Kim[4*], and Kyeong-Keun Choi[5]

[1]Department of Engineering in Energy and Applied Chemistry Silla University, Busan 617-736 (Republic of Korea)

[2]Department of Electrical Engineering Korea Advanced Institute of Science and Technology (KAIST), 291 Daehak-ro, Yuseong-gu, Daejeon 305-701 (Republic of Korea)

[3]Advanced Materials Engineering Korea Polytechnic University, Jungwang dong Shihung 429-793 (Republic of Korea)

[4]Institute of NT.IT fusion technology, Ajou university, Worldcup ro 260, Youngtong gu Suwon 16499 (Republic of Korea)

[5]National Center for Nanomaterials Technology (NCNT) San 31, Hyoja-Dong, Nam-Gu, Pohang 790-784 (Republic of Korea)

.

1. Sheet resistance of spin-coated i-RGO versus vacuum pressure

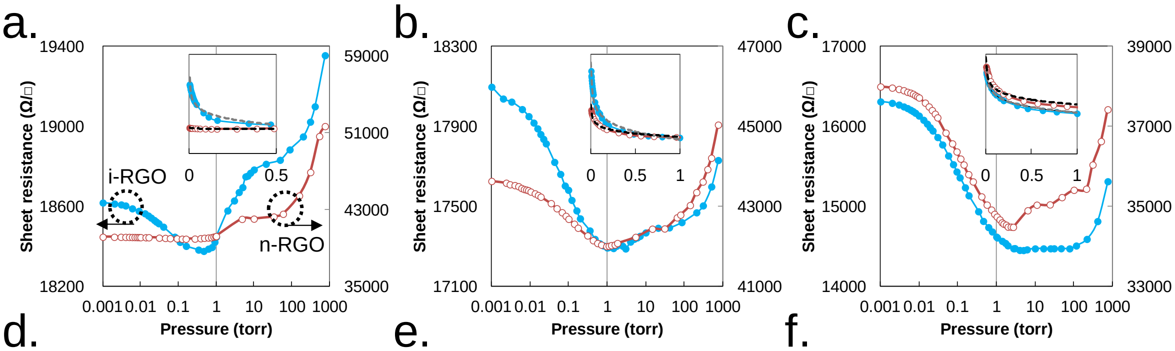

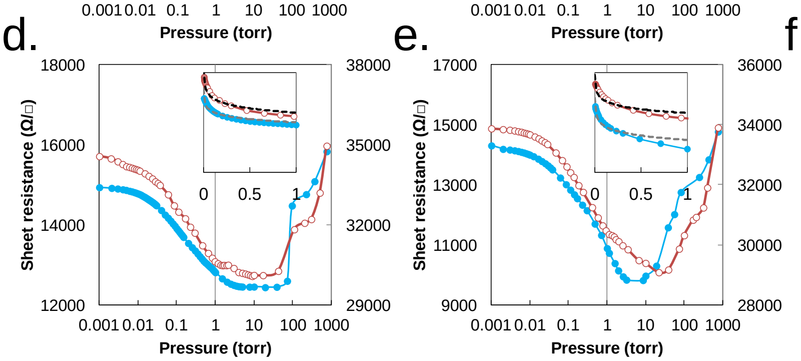

f.

Temp. (°C)	n-RGO Fitting Eq.	i-RGO Fitting Eq.
30	$R = 10631P^2 - 2928P + 40111$	$R = 11986P^2 - 3152.4P + 18607$
50	$R = -277.8\ln(P) + 42022$	$R = -134.7\ln(P) + 17271$
100	$R = -554.3\ln(P) + 34947$	$R = -290.4\ln(P) + 14691$
150	$R = -656.2\ln(P) + 30943$	$R = -392.4\ln(P) + 12940$
200	$R = -555.7\ln(P) + 30934$	$R = -510.7\ln(P) + 11499$

Figure S1. Sheet resistance of spin-coated i-RGO (with a PVA/GO weight ratio of 1) and normal RGO (n-RGO) as pressure is elevated from 10^{-3} torr to ambient air pressure; (a) 30 °C, (b) 50 °C, (c) 100 °C, (d) 150 °C, and (e) 200 °C. (f) A table of fitting equations used to calculate errors in the pressure readings below 1 torr. Insets are enlarged graphs with fitting curves plotted on a linear scale. The sheet resistances were measured at 1.67 s intervals as a function of increasing pressure in a vacuum under a constant leakage of approximately 3×10^{-3} torr/min.

2. Sensitivity and reproducibility of pressure reading from spin-coated i-RGO

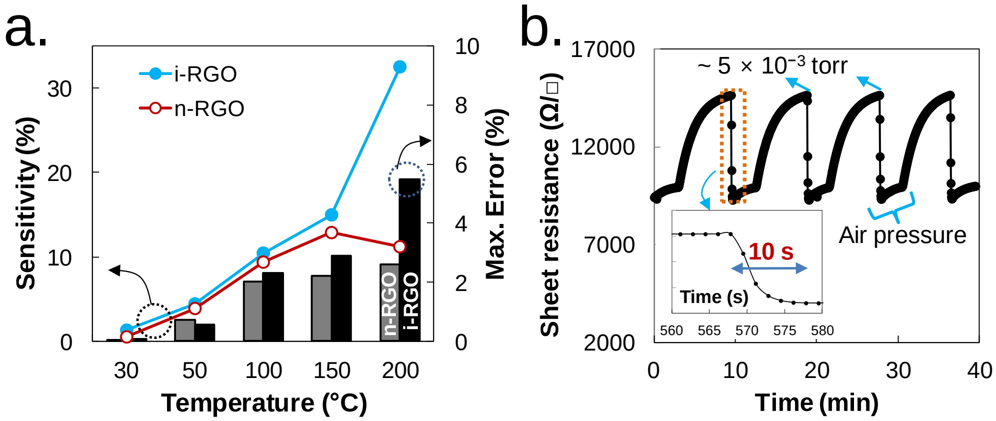

Figure S2. Sensitivity, maximum error, and reproducibility of pressure readings between 0.001 and 1 torr; (a) Sensitivities of RGO samples and maximum errors at measurement temperature from 30 to 200 °C, (b) Repeated measurement of sheet resistance in spin-coated i-RGO at 200 °C in the vacuum range between ~ 5×10^{-3} and ambient air pressure. Note that the percentage error was calculated as $100 \times |\Delta R| / R_{fitting}$ (where $\Delta R = R_{real} - R_{fitting}$ at a given pressure).

3. Theoretical description of resistance behavior in spin-coated i-RGO versus vacuum pressures

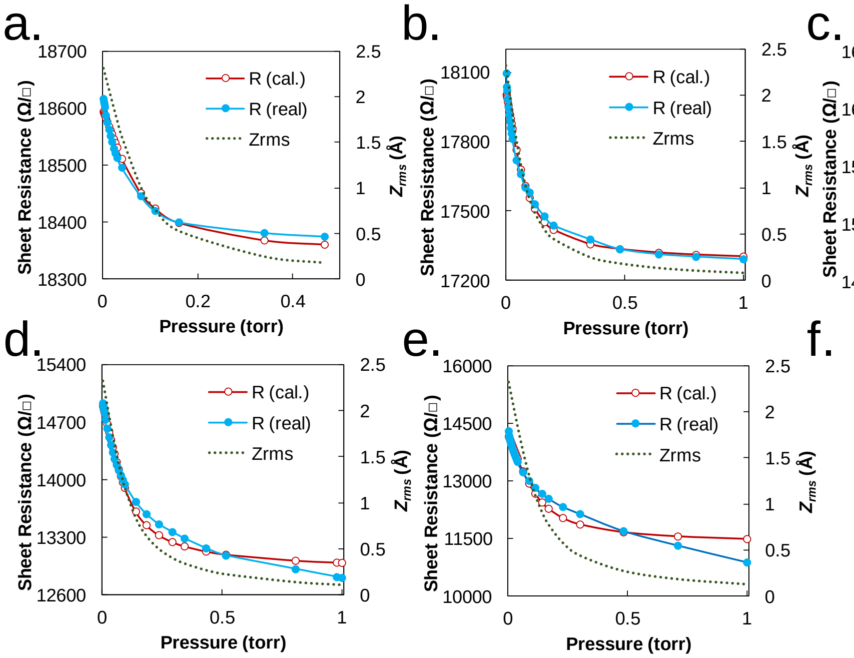

T (℃)	a	b
30	18341.4	109.12
50	17277.7	313.28
100	14666.7	681.79
150	12896.1	865.07
200	11338.8	1218.6

Figure S3. Theoretically calculated standard deviations of the z axis (Z_{rms}) and resistance of the spin-coated i-RGO film as the pressure is increased from 10^{-3} to 1 torr; (a) at 30 ℃, (b) at 50 ℃, (c) at 100 ℃, (d) at 150 ℃, and (e) at 200 ℃. (f) Table of fitting parameters used for equation (2) (R= a + b Z_{rms}). Note that the calculated resistances are compared with the experimental data in Figure S1.

The suggested hypothesis of LVW interaction is theoretically examined in the low pressure regime, with the assumptions that the parallel horizontal layers of hexagonal graphene lattice have a side length of 1.42 Å connected vertically by polymer pillars of 5 nm radius and the distance between two layers is 6.8 Å. For a pressure of 1 torr and a temperature of 300 K, the number density of air molecules is quite small, about one per 30 nm side cube. Therefore, the pressure equilibrium condition of the graphene sheets seem to play a role in resistance changes at low pressure, rather than the direct action of air molecules inside graphene. In this regime the pres-

sure on each atom (~ 1.89×10^{-28} J) is small compared with the effects, for example, thermal energy (~ 4.14×10^{-21} J) and VDW minimum energy (~ 3.86×10^{-22} J). Hence, the manifested pressure effect is likely to be a macroscopic effect. For the theoretical calculation, one graphene layer between parallel layers is considered, with the normal direction of the layer along the Z axis, and the average position of the layer in this axis is Z. Supposing that the distances between upper and lower layers are the same, the Z value becomes zero. In smaller length scale (horizontal layer diameter ~5 nm) Z can fluctuate by a few Å since, locally, the layer can be attracted to lower or upper layers by van der Waals attraction. At a larger scale (horizontal layer diameter ~ 1 μm) local parts of the layers can be randomly attracted to the upper or lower layers. If the deformation is symmetric about the Z direction, we can assume that Z = 0 is the average equilibrium position of the layer. The potential energy of the layer along the Z plane can become complex, depending on the size of the layer. In the regime where pressure differences cause a visible change, one can assume that the average position of layer Z changes by ΔZ, since the layer has higher energy by $\mathrm{P}\,\Delta\mathrm{V} = \mathrm{P}\,\mathrm{A}\,|\Delta\mathrm{Z}|$ (P is pressure and A is the area of the layer). The probability density also changes by a factor $\exp(-\beta\,\mathrm{P}\,\mathrm{A}\,|\Delta\mathrm{Z}|)$. The resistance is related to mobilities of the charge carriers. When Z has an appreciable standard deviation, the carriers should travel a longer path with potential barriers. We assume that the resistance has a linear relation with the standard deviation of Z. Since the average of Z is 0, the standard deviation of Z is just Z_{rms}. Using the above partition function factor, the following equation can be obtained:

$$Z_{rms}(T, A, P) = \left(\frac{\int dz\ z^2 \exp(-\beta\,\mathrm{P}\,\mathrm{A}\,|\mathrm{Z}|)}{\int dz\ \exp(-\beta\,\mathrm{P}\,\mathrm{A}\,|\mathrm{Z}|)}\right)^{1/2} \quad (1)$$

With the given experimental data in Figure 2 and a suitable value for A, the (P vs Z_{rms}) graph correlates with the (P vs R) graph assuming:

$$R = a + b\, Z_{rms} \qquad (2)$$

(a and b in equation (2) are T and A dependent)

Figure S3 shows (P vs R) and (P vs Z_{rms}) graphs in comparison with the experimental data in Figure S1. A is set as 5.25×10^{-12} m^2, which corresponds to a square with a side length of 2.29 μm. The fitting parameters are a, b, and Z_{rms} in units of Å. As seen in Figure 6, the theoretical description with A is consistent matches with the experimental data.

4. A schematic of reaction-based self-assembly for the preparation of i-RGO

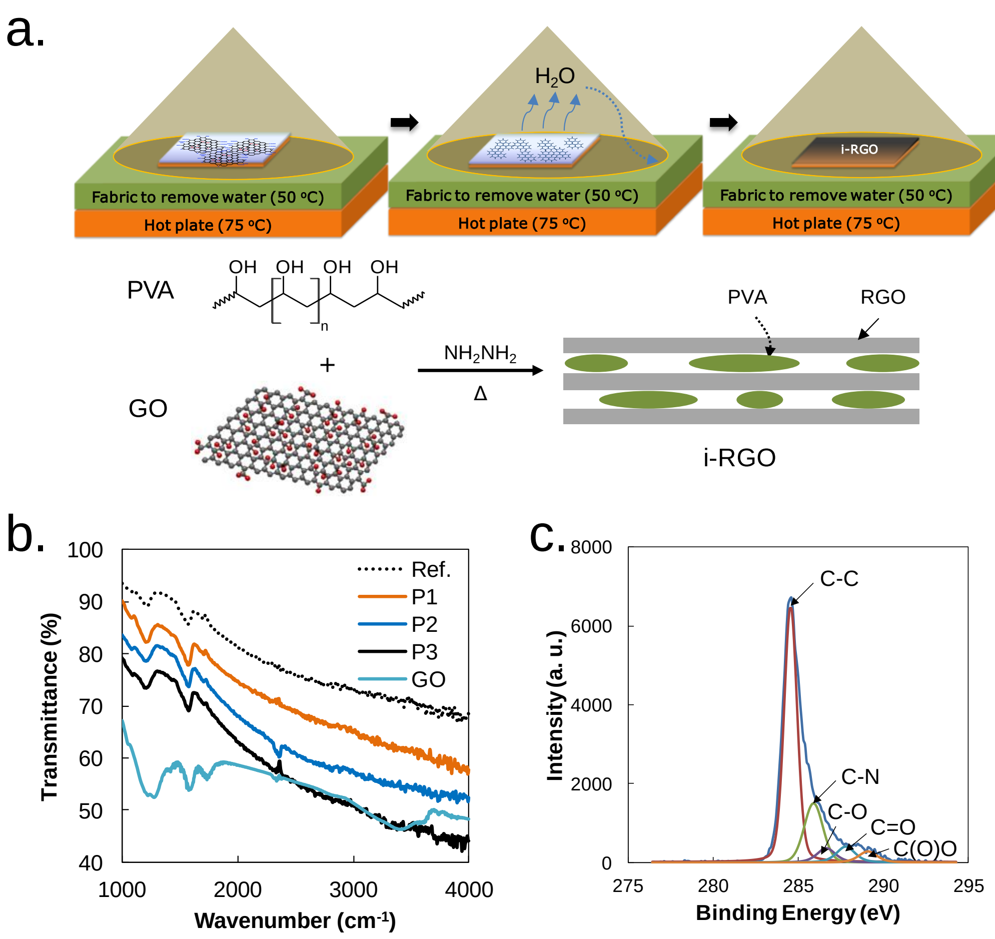

Figure S4. (a) A schematic of reaction-based self-assembly of i-RGO, (b) FT-IR spectra of the samples after heat-treatment at 120 °C for 10 min, (c) XPS spectrum of sample Ref. RGO formed by the RSA method.

5. A test device using patterned ITO electrodes

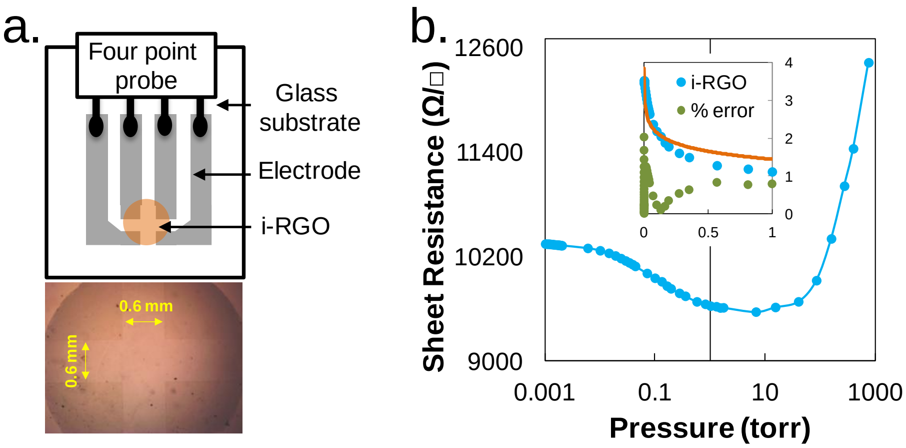

Figure S5. Properties of a pressure sensor using ITO electrodes; (a) A diagram of the test device structure and a microscope image of the active area, (b) Results of sheet resistance measurements in the device with increasing pressure.

6. Sheet resistance of RSA i-RGO at high vacuum pressures

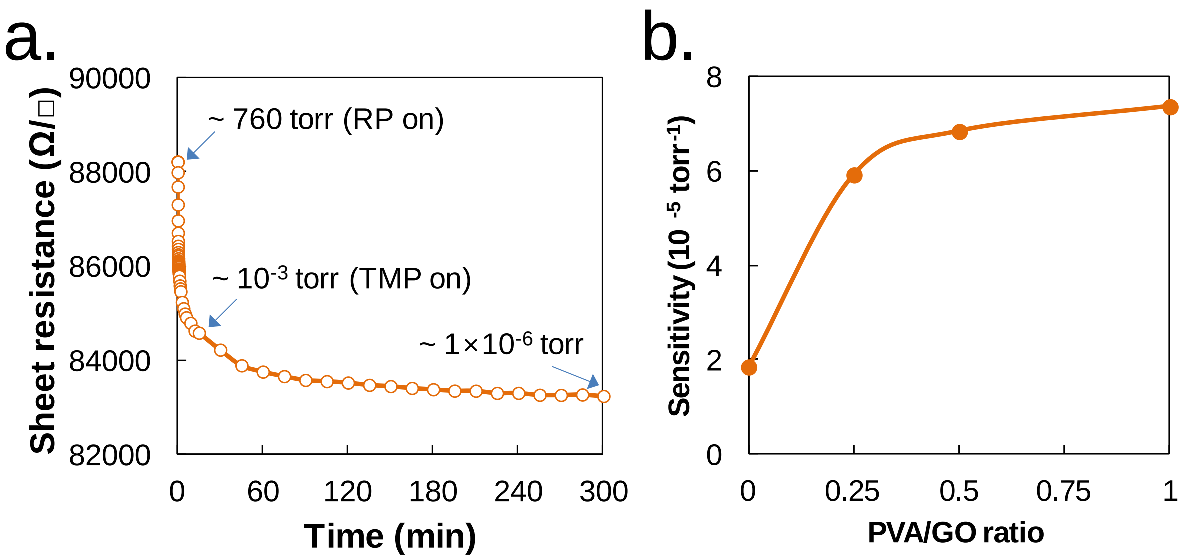

Figure S6. (a) Sheet resistance of RSA i-RGO (P3) with increasing vacuum pressure from ambient air pressure to approximately 10^{-6} torr at 20 °C (RP and TMP indicate the rotary and turbo molecular pumps, respectively), (b) Sensitivity of pressure readings within the pressure range used in (a). Note that the sensitivity is calculated as $\Delta R/R_{max} \cdot P$. Here, $P = |P_{max} - P_{min}|$ and $\Delta R = |R_{max} - R_{min}|$, where R_{max} and R_{min} are the maximum and minimum sheet resistance, respectively, in the pressure range between 10^{-6} and 760 torr.
